# Machine Learning Analysis of Engagement Behaviors in Older Adults With Dementia Playing Mobile Games: Exploratory Study

**DOI:** 10.2196/54797

**Published:** 2025-03-03

**Authors:** Melika Torabgar, Mathieu Figeys, Shaniff Esmail, Eleni Stroulia, Adriana M Ríos Rincón

**Affiliations:** 1 Department of Occupational Therapy, University of Alberta, Edmonton, AB, Canada; 2 Department of Computing Science, University of Alberta, Edmonton, AB, Canada

**Keywords:** dementia, gaming, engagement, cognition, machine learning, games, cognitive, screening, classification, Alzheimer disease, gerontology, geriatric, older adult, elderly, aging

## Abstract

**Background:**

The prevalence of dementia is expected to rise with an aging population, necessitating accessible early detection methods. Serious games have emerged as potential cognitive screening tools. They provide not only an engaging platform for assessing cognitive function but also serve as valuable indicators of cognitive health through engagement levels observed during play.

**Objective:**

This study aims to examine the differences in engagement-related behaviors between older adults with and without dementia during serious gaming sessions. Further, it seeks to identify the key contributors that enhance the effectiveness of machine learning for dementia classification based on engagement-related behaviors.

**Methods:**

This was an exploratory proof-of-concept study. Over 8 weeks, 20 older adults, 6 of whom were living with dementia, were enrolled in a single-case design study. Participants played 1 of 4 “Vibrant Minds” serious games (Bejeweled, Whack-A-Mole, Mah-jong, and Word-Search) over 8 weeks (16 30-min sessions). Throughout the study, sessions were recorded to analyze engagement-related behaviors. This paper reports on the analysis of the engagement-related behaviors of 15 participants. The videos of these 15 participants (10 cognitively intact, 5 with dementia) were analyzed by 2 independent raters, individually annotating engagement-related behaviors at 15-second intervals using a coding system. This analysis resulted in 1774 data points categorized into 47 behavior codes, augmented by 54 additional features including personal characteristics, technical issues, and environmental factors. Each engagement-related behavior was compared between older adults living with dementia and older adults without dementia using the *χ*² test with a 2×2 contingency table with a significance level of .05. Codes underwent one-hot encoding and were processed using random forest classifiers to distinguish between participant groups.

**Results:**

Significant differences in 64% of engagement-related behaviors were found between groups, notably in torso movements, voice modulation, facial expressions, and concentration. Including engagement-related behaviors, environmental disturbances, technical issues, and personal characteristics resulted in the best model for classifying cases of dementia correctly, achieving an *F*_1_-score of 0.91 (95% CI 0.851‐0.963) and an area under the receiver operating curve of 0.99 (95% CI 0.984‐1.000).

**Conclusions:**

Key features distinguishing between older adults with and without dementia during serious gameplay included torso, voice, facial, and concentration behaviors, as well as age. The best performing machine learning model identified included features of engagement-related behavios, environmental disturbances, technical challenges, and personal attributes. Engagement-related behaviors observed during serious gaming offer crucial markers for identifying dementia. Machine learning models that incorporate these unique behavioral markers present a promising, noninvasive approach for early dementia screening in a variety of settings.

## Introduction

The world’s population is aging at an unprecedented rate, which will impact the prevalence rate of older adults living with dementia. The World Health Organization projects that there will be over 132 million people living with dementia by 2050 [1]. Dementia not only affects the individual’s cognitive functioning but also creates several challenges for families, caregivers, and health care systems. Moreover, the economic burden of dementia is burdensome, with global estimated economic costs of nearly US$1 trillion in 2016 [2].

Dementia encompasses a group of disorders characterized by a gradual decline in memory, reasoning, communication, and daily functioning, typically due to brain damage. Alzheimer disease (AD) is the most common form, followed by vascular dementia and dementia with Lewy bodies [3]. Cognitive impairments, such as dementia, are typically assessed and monitored by health care professionals during routine clinical examinations or when an older adult or their family members identify symptoms of cognitive decline and seek assistance [3]. Limitations of current screening have spurred research into the development of alternative, more naturalistic methods to monitor for cognitive decline. Early identification and the prevention of cognitive decline are critical in reducing the burden of dementia on individuals, families, health care systems, and larger economies and societies.

The use of machine learning (ML) in assessing cognitive decline is an emerging field of study. A recent scoping review by Tolks et al [4] identified a limited evidence base (16 articles) on the use of artificial intelligence in health-related serious games, predominantly using ML methods. Among these, only 2 studies focused on mild cognitive impairment, with none specifically targeting dementia. Beyond serious gaming, Bayat et al [5] developed an ML model with a 91% accuracy in identifying older individuals with and without preclinical AD (the phase that occurs prior to the onset of symptoms) by analyzing driving behaviors recorded through in-vehicle global positioning system data loggers. Similarly, Padhee et al [6] built an ML model with an 81% accuracy rate to distinguish individuals with AD from those without AD based on verbal utterances while describing a picture.

Computer serious games performance has been used to distinguish dementia and mild cognitive impairment in older adults using ML techniques [7-9]. While using game performance and ML techniques to distinguish dementia in older adults can have some potential benefits, there are also disadvantages to consider. First, there is variability in the computer games designed to assess cognitive abilities in aspects such as the cognitive domains targeted, topics or themes of the game, levels of difficulty, and protocols. This variability prevents the generalization of the results, limiting the performance of an ML algorithm to a specific game and under specific conditions. Second, older adults with dementia may have varying levels of interest, motivation, and familiarity with the computer game being used for assessment. Thus, assessments that rely exclusively on game performance can lead to unreliable or biased results. In our study, we explore engagement behaviors instead of game performance to distinguish dementia in older adults.

Engagement is a complex construct that has behavioral, cognitive, and affective dimensions [10]. The behavioral dimension includes effort and task persistence, whereas the cognitive dimension includes attention and concentration. The affective dimension includes valence, arousal, as well as discrete emotions such as curiosity and interest [11]. The multifaceted nature of engagement suggests that behaviors related to engagement could potentially provide insights into the presence and progression of dementia.

This study aims to contribute to the ongoing efforts to develop new and objective methods of assessing cognitive decline in older adults. In doing so, this study tested two hypotheses: (1) there are significant differences in engagement-related behaviours of older adults living with dementia and those without dementia while playing mobile games and (2) a dataset of engagement-related behaviours, along with other characteristics such as personal characteristics, environmental disturbances, and technical issues, generates distinct patterns for the presence of dementia in older adults while playing mobile games.

## Methods

### Study Design

In total, 20 older adults, including 6 individuals living with dementia, participated in a single-case design study [12]. This paper focuses on analyzing engagement-related behaviors observed in 15 participants who were video-recorded during their game sessions.

### Participants

Participants were eligible if they were 60 years or older, cognitively intact or self-reported as having dementia, and able to attend the scheduled sessions. All participants were recruited from the community, and the study was conducted in 2 settings: a community organization providing support to individuals with dementia and their families and a supportive living facility, both located in Edmonton, Alberta, Canada [12]. Each participant was randomly assigned to 1 of 4 “Vibrant Minds” [13] mobile games developed for cognitive assessment and intervention. The method used for random allocation involved a lottery-style draw, a well-recognized approach to ensure randomization. Each participant randomly selected a card from a set of 4 cards, each featuring the logo of one of the “Vibrant Minds” mobile games (Bejeweled, Whack-A-Mole, Mah-jong, or Word-Search). To maintain blinding during the selection, the cards were placed face down with identical backs, ensuring that participants were unaware of the game assignment prior to selection. This approach was administered and supervised by a member of the research team to ensure fairness and adherence to the protocol. Once assigned, participants continued playing the same game throughout the 8-week study, which included 16 sessions lasting 30 minutes each. These games were designed to provide cognitive stimulation for older adults and were played on a 10-inch tablet in each session. Sessions took place in a designated room where participants were seated at a table. They were held twice weekly, typically on the same days and at the same time whenever feasible. While participants completed the activities individually, they shared the same room. Each session was supervised by 2-5 research assistants who prepared the space, materials, and equipment; provided instructions; and assisted participants as needed. Sessions lasted 30 minutes, with participants receiving consistent verbal instructions: “We prepared these activities for you. We want you to do these for 30 minutes.” Research assistants collected demographic data (eg, age, gender, education, technology literacy, and experience with serious games) and administered cognitive measures (eg, Montreal Cognitive Assessment [14]) at each phase of the single-case design. Cognitive measures assessed the intervention’s potential impact on cognitive functions, with results reported elsewhere [12].

### Data Source

#### Overview

**Figure 1.**
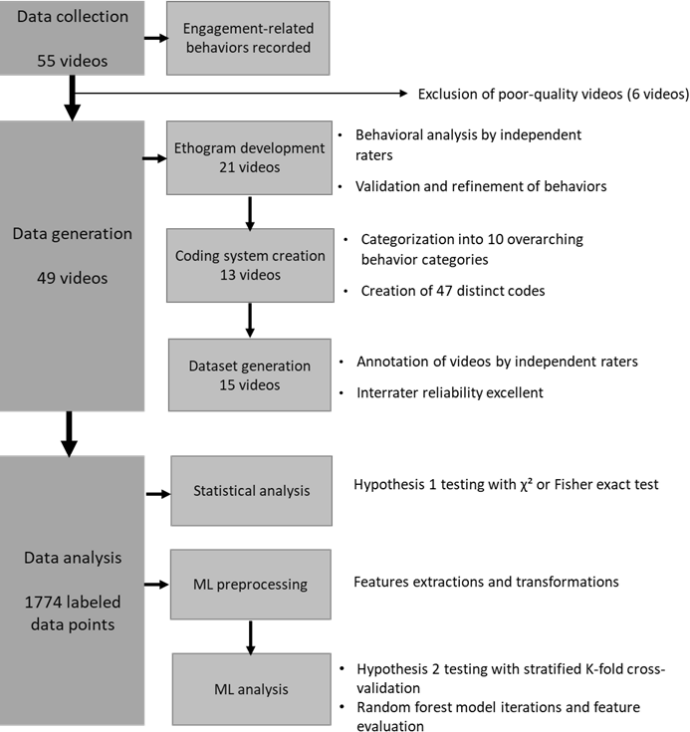
Study flow chart. ML: machine learning.

Figure 1 illustrates the study phases. Throughout the study, engagement-related behaviors were recorded for all participants during at least 1 session, resulting in 55 videos. Participants for each session were randomly selected using a lottery-style draw, and each recording lasted 30 minutes. The video footage captured each participant’s face, trunk, and upper limbs [15]. In total, 6 videos were excluded due to poor quality, leaving 49 usable recordings.

Our approach consisted of 3 phases: (1) the development of an ethogram (a detailed description and analysis of behaviors), (2) the creation of a coding system (a structured framework for categorizing and labeling behaviors to enable systematic analysis), and (3) the generation of the dataset of engagement-related behaviors. This methodology has been applied in prior research involving individuals living with dementia [16]. A total of 34 videos (70% of the 49 usable recordings) were used during the first 2 phases to ensure a comprehensive and rigorous foundation for subsequent analyses [15].

#### Ethogram Development

In total, 2 independent raters analyzed video sequences to identify and describe behaviors indicative of engagement, disengagement, or neutrality. A second set of raters reviewed the identified behaviors to validate those with clear relevance to engagement. Ambiguous behaviors were excluded through team discussions to ensure clarity and consistency.

#### Coding System Creation

A separate researcher reviewed the validated behaviors and organized them into 10 overarching categories: gaze, eyes, head, torso, limbs, face, voice, gameplay, concentration, and breath. Within each category, behaviors were grouped by similarities, resulting in 47 distinct codes that formed a structured framework for behavior analysis.

#### Dataset Generation

The remaining 15 videos (30%) were analyzed to create a dataset of engagement-related behaviors. These videos represented a subset of 15 participants, comprising 10 cognitively intact older adults and 5 individuals with mild-to-moderate dementia. In total, 2 independent raters annotated the videos, identifying engagement-related behaviors at 15-second intervals using the coding system. This process resulted in a dataset with 1774 labeled data points. Interrater reliability for the coding system was excellent (κ=0.87) [15].

All raters underwent training in engagement theory and behavior analysis, led by a senior member of the research team. Importantly, the raters were blinded to the demographic information of participants in the videos, including their dementia status.

### Ethical Considerations

Potential participants and their caregivers were given recruitment flyers with the research team’s contact details. Cognitively healthy older adults, individuals with mild dementia, or their substitute decision-makers contacted the research team to express interest. A team member then scheduled in-person or telephone meetings to explain the study and confirm. Written consent was obtained from participants or their substitute decision-makers. The University of Alberta Research Ethics Board reviewed and approved this study (Pro00069138). Three times during the study, participants were given a coffee shop gift card, ranging in value from CAD $10 to $25 (US $6.99 to $17.47). All personal identifying information was removed (deidentified) before analysis.

### ML Preprocessing

The input data encompassed 54 features, which incorporated the 47 engagement-related behaviors along with personal characteristics (ie, age, gender, highest level of education, technology literacy, and prior experience with serious games), environmental disturbances (ie, any disruption during the game session), and technical issues. Age was transformed into a categorical variable using the visual binning method in SPSS (version 23; IBM). A one-hot encoding method was used to convert the categorical codes into binary values.

### Statistical Analysis

To test hypothesis 1, each engagement-related behavior was compared between older adults living with dementia and older adults without dementia using the *χ*² test with a 2×2 contingency table with a significance level of .05. For expected frequencies of less than 5 occurrences, Fisher exact test was employed [17]. The analyses were performed using SPSS Statistics (version 23.0; IBM Corp.).

### ML Analysis

To test hypothesis 2, the preprocessed dataset underwent stratified K-fold cross-validation [18], due to the ratio of older adults living with dementia versus older adults without dementia being 0.33 [19]. The dataset was divided into 4 different folds, with the model trained on three folds and tested on the remaining fold in each iteration. The training dataset constituted 75% of the data, and the test dataset comprised 25%. Results were obtained by averaging values from test samples across 4 iterations.

A random forest (RF) classifier, a supervised ML model, was used to distinguish between relevant engagement-related behaviors of older adults with and without dementia. In total, 4 RF models were trained, each with different sets of input variables. The models were trained on the parameters listed below, and the best-performing model was chosen: (1) engagement-related behavior features; (2) engagement-related behaviors features and personal features; (3) engagement-related behaviors features, environmental disturbance features, and technical issues features; (4) engagement-related behavior features, environmental disturbance features, technical issues features, and personal features.

Precision, recall, and accuracy (*F*_1_) scores with 95% confidence intervals were calculated and compared across the 4 models [20]. To evaluate the performance of each model, a receiver operating curve was generated to visualize the true positive rate against the false positive rate at various thresholds, and the area under the receiver operating curve (AUC) was computed for each model. A high AUC (ranging from 0 to 1) indicates a model with a good fit in distinguishing between conditions [21,22].

The feature importance method was used to identify older adults with dementia from those without dementia [23]. This method assigns a score to each feature, with higher scores indicating greater importance for the machine’s prediction. The RF Gini index was used to calculate the decrease in node impurity, which measures how the features of a dataset should be divided into nodes in constructing a decision tree [24,25]. The relevant features with a score of at least 0.05 in a decrease in mean impurity were selected, as these relevant features are important for identifying older adults with or without dementia.

## Results

### Demographics

Older adults without dementia were, on average, 3.1 years older than participants with dementia. The ratio of women to men was equal in both groups. A high school diploma was the most common highest level of education for older adults in both groups (5/15, 33%). Computers were the most used information and communication technologies by older adults without dementia (6/10, 60%). Additionally, both groups had similar previous experiences with serious gaming (8/15, 60%), as shown in Table 1.

**Table 1.** Participant demographics. Participants living with dementia were on average 76.6 (SD 6.8) years old. Participants without dementia were on average 79.7 (SD 8.8) years old. The mean Montreal Cognitive Assessment (MoCA) [12] was 21.75 (SD 7.48); mean MoCA for older adults living with dementia was 13.60 (SD 7.82); mean MoCA older adults without dementia was 25.45 (SD 3.36).

Variable		Dementia (n=5), n (%)	Without dementia (n=10), n (%)
Sex			
	Female	3 (60)	6 (60)
	Male	2 (40)	4 (40)
Highest educational level			
	Elementary school	0 (0)	1 (10)
	High school diploma	2 (40)	3 (30)
	Trade or vocational training	1 (20)	0 (0)
	College diploma	1 (20)	2 (20)
	Bachelor’s degree	1 (20)	3 (30)
	Master’s degree	0 (0)	1 (10)
Technology literacy (ICT^a^ used)			
	Computer	2 (40)	6 (60)
	Computer and tablet	2 (40)	0 (0)
	Computer and smartphone	0 (0)	1 (10)
	Computer, tablet, and smartphone	1 (20)	3 (30)
Previous experience with serious games		3 (60)	6 (60)

a ICT: information and communication technology.

### Descriptive Analysis of Engagement-Related Behaviors in Older Adults

The descriptive analysis revealed that the most common behaviors, occurring over 70% of the time in both groups, were related to gaze (ie, gaze toward game) (1767/1774, 99.6%), eyes (ie, scanning behavior) (1760/1774, 99.2%), head (ie, scanning behavior) (1765/1774, 99.5%), face (ie, neutral expression) (1603/1774, 90.4%), gameplay (ie, physical interaction with screen) (1755/1774, 99%), and concentration (ie, not distracted by external stimuli) (1611/1774, 90.8%).

Notably, older adults without dementia exhibited lean-forward behaviors and eyebrow movement more frequently compared to those with dementia. In contrast, older adults with dementia demonstrated higher frequencies of upright posture, voiced utterance, and playing while doing something else compared to those without dementia.

Certain behaviors had zero frequencies in older adults with dementia. Examples are engagement behaviors such as saccadic eye movements and keeping up with the game as well as disengagement behaviors such as slouched posture or yawning.

### Differences in Behavior Frequencies Across Dementia Status

**Figure 2.**
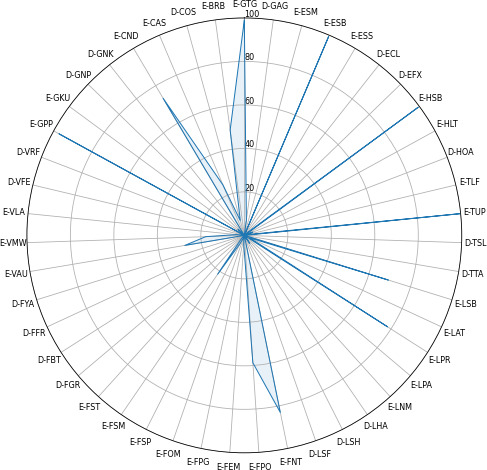
Radar plot of the behavioral frequencies of older adults with dementia. D-COS: stopping play to attend to another stimulus; D-ECL: closed eyes; D-EFX: eyes fixed on point; D-FBT: angry expression; D-FFR: frowning; D-FGR: pained expression; D-FYA: yawning; D-GAG: gaze away from the game; D-GNK: not keeping up with the game; D-GNP: not physically interacting with the screen; D-HOA: head oriented away from the display; D-LHA: play hand away from device; D-LSH: play hand movements unrelated to the game; D-LSF: smacking face; D-TSL: slouched posture; D-TTA: turned away from the game; D-VFE: frustrated exclamation; D-VRF: refusal; E-BRB: rhythmic breathing; E-CAS: playing while doing something else; E-CND: not distracted by external stimuli; E-ESB: scanning behaviors; E-ESM: saccadic eye movements; E-ESS: squinting at screen; E-FEM: eyebrow movement; E-FNT: neutral expression; E-FOM: open mouth; E-FPG: playful grimace; E-FPO: lip behavior; E-FSM: smiling; E-FSP: surprised expression; E-FST: tongue behavior; E-GKU: keeping up with the game; E-GPP: physical interaction with the screen; E-GTG: gaze toward game; E-HLT: head learning toward game; E-HSB: scanning behavior; E-LAT: adjust the position of the tablet; E-LNM: non-play hand at mouth; E-LPA: play arm position adjustment; E-LPR: play hand ready; E-LSB: limbs scanning behavior; E-TLF: leaning forward; E-TUP: upright posture; E-VAU: voiced utterance; E-VLA: laughing; E-VMW: voiceless utterance.

A comparison of the frequencies of the 47 engagement-related behaviors, along with their respective statistics and *P* values, is presented in Table S1 in Multimedia Appendix 1. A total of 30 out of 47 (64%) engagement-related behaviors showed statistically significant differences in frequencies between older adults living with dementia and those without dementia. Among the different behavioral categories, the face category exhibited the highest number of behaviors, with 8 out of 12 (66.6%), showing significant differences between the 2 groups. Figures2  3 display graphical representations detailing the variance in behavioral frequencies among older adults in each group, depicted as distinctive behavioral fingerprints.

**Figure 3.**
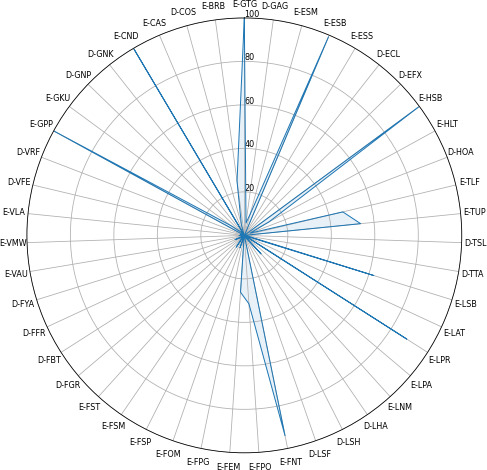
Radar plot of the behavioral frequencies of older adults without dementia. D-COS: stopping play to attend to another stimulus; D-ECL: closed eyes; D-EFX: eyes fixed on point; D-FBT: angry expression; D-FFR: frowning; D-FGR: pained expression; D-FYA: yawning; D-GAG: gaze away from the game; D-GNK: not keeping up with the game; D-GNP: not physically interacting with the screen; D-HOA: head oriented away from the display; D-LHA: play hand away from device; D-LSH: play hand movements unrelated to the game; D-LSF: smacking face; D-TSL: slouched posture; D-TTA: turned away from the game; D-VFE: frustrated exclamation; D-VRF: refusal; E-BRB: rhythmic breathing; E-CAS: playing while doing something else; E-CND: not distracted by external stimuli; E-ESB: scanning behaviors; E-ESM: saccadic eye movements; E-ESS: squinting at screen; E-FEM: eyebrow movement; E-FNT: neutral expression; E-FOM: open mouth; E-FPG: playful grimace; E-FPO: lip behavior; E-FSM: smiling; E-FSP: surprised expression; E-FST: tongue behavior; E-GKU: keeping up with the game; E-GPP: physical interaction with the screen; E-GTG: gaze toward game; E-HLT: head learning toward game; E-HSB: scanning behavior; E-LAT: adjust the position of the tablet; E-LNM: non-play hand at mouth; E-LPA: play arm position adjustment; E-LPR: play hand ready; E-LSB: limbs scanning behavior; E-TLF: leaning forward; E-TUP: upright posture; E-VAU: voiced utterance; E-VLA: laughing; E-VMW: voiceless utterance.

### Random Forest Models

**Figure 4.**
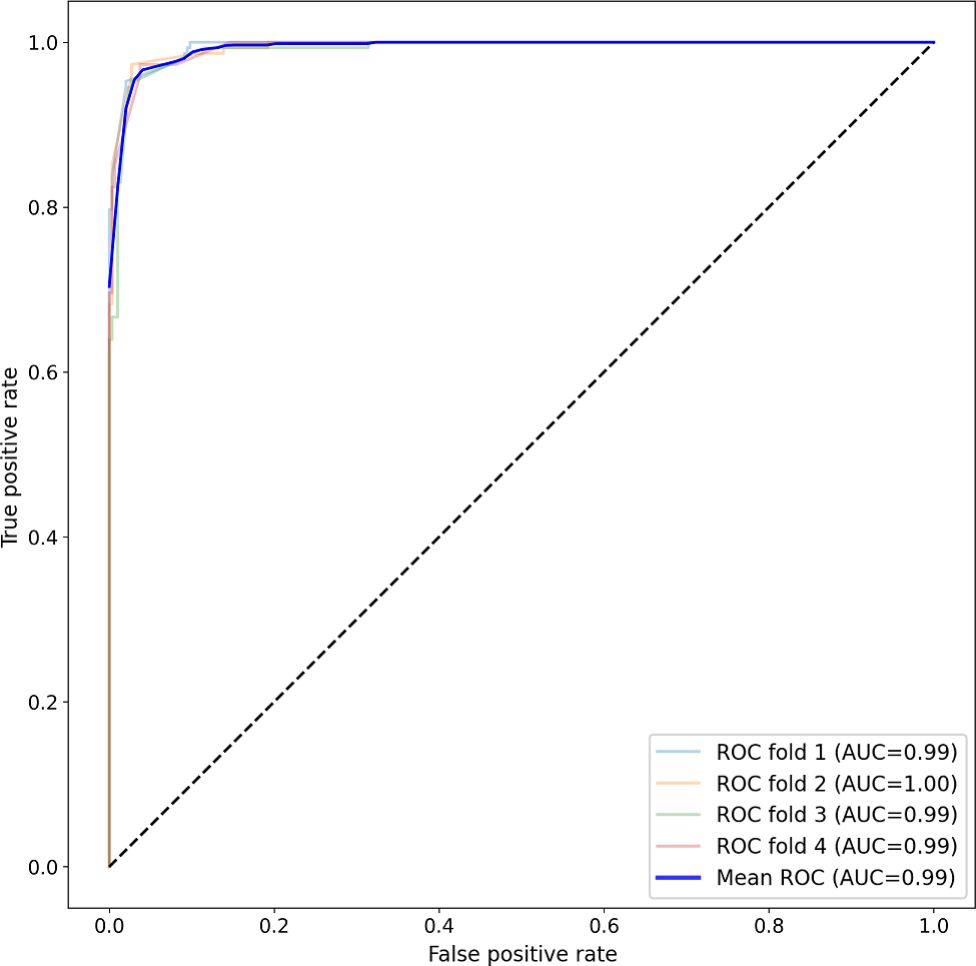
The AUC for engagement-related behaviors, personal features, environmental disturbance, and technical issues. ROC: receiver operating curve; AUC: area under the receiver operating curve.

In total, 4 different RF models were trained and evaluated using *F*_1_-scores to identify the best model. The higher *F*_1_-score indicated a better balance between precision and recall, and the higher AUC indicated superior performance in classifying between the 2 groups. Table 2 highlights the trained model performances by variable. Model 4 achieved the highest *F*_1_-score (91%) and AUC (99%) among all the models, making it the best suitable model (see Figure 4).

**Table 2.** Model performance by input variables.

Model	Input variables	Precision (95% CI)	Recall (95% CI)	*F*_1_-score (95% CI)	AUC^a^ (95% CI)
1	Engagement-related behaviors	0.69 (0.654‐0.715)	0.93 (0.843‐1.000)	0.78 (0.770‐0.813)	0.96 (0.939‐0.970)
2	Engagement-related behaviors and personal features	0.81 (0.780‐0.839)	0.98 (0.958‐1.000)	0.88 (0.872‐0.902)	0.99 (0.984‐1.000)
3	Engagement-related behaviors, environmental disturbance, and technical issues	0.68 (0.634‐0.730)	0.94 (0.875‐1.000)	0.79 (0.774‐0.815)	0.96 (0.949‐0.965)
4	Engagement-related behaviors, environmental disturbance, technical issues, and personal features	0.85 (0.724‐0.990)	0.97 (0.930‐1.000)	0.91 (0.851‐0.963)	0.99 (0.984‐1.000)

a AUC: area under the receiver operating curve.

### Importance of Features in Identifying Older Adults With Dementia

**Figure 5.**
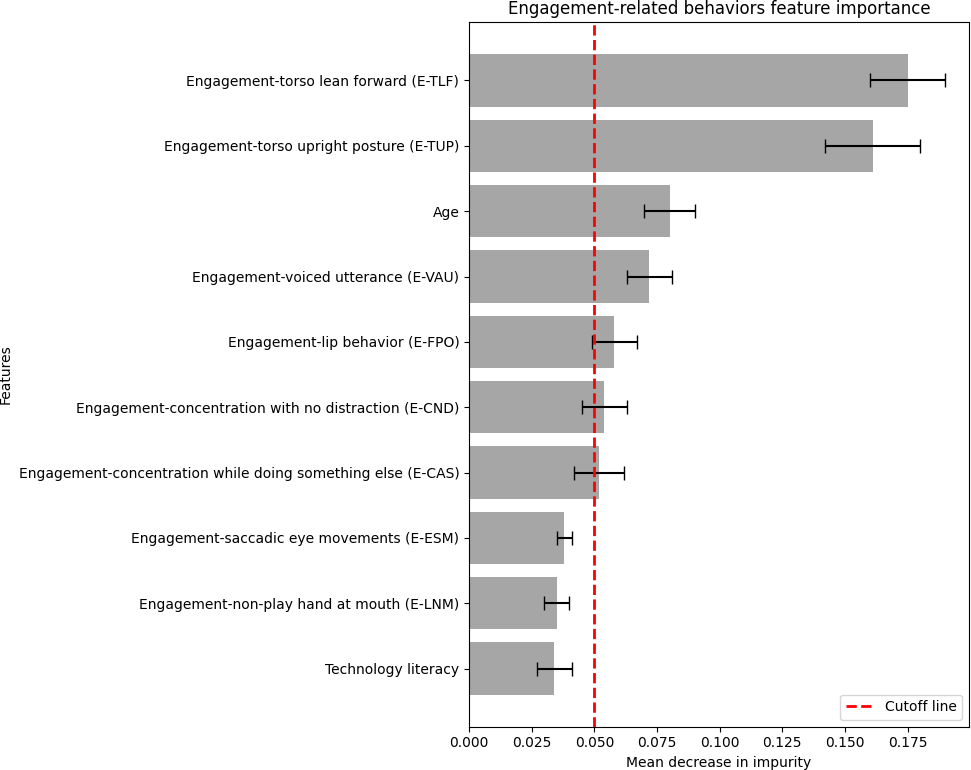
A bar graph representing the feature importance by mean decrease in data impurity and their confidence intervals. Behaviors with higher impurity were considered more relevant.

Ranked importance of features in identifying older adults with dementia was quantified by impurity (disorder within the data). The measure of the mean decrease in impurity, visualized in Figure 5, demonstrated that behaviors with higher impurity were considered more relevant, while behaviors with decreased impurity were deemed less important or irrelevant to older adults with dementia. The engagement-related behaviors of torso lean forward and torso upright posture were found to be the most relevant in distinguishing between older adults with and without dementia. Age was also determined as a relevant factor. The feature importance analysis highlights key behavioral indicators that differentiate older adults with and without dementia. Six primary behavioral categories—Face, Concentration, Torso, Voice, Eyes, and Limbs—were identified, each containing specific behaviors relevant to distinguishing between the two groups. Certain features, such as lip behavior (Face), playing while doing something else (Concentration), upright posture and E-TLF (Torso), and voiced utterance (Voice), were more commonly associated with older adults living with dementia. In contrast, features like not distracted by external stimuli (Concentration), and leaning forward (Torso), saccadic eye movements (Eyes), and non-play hand at mouth (Limbs) were primarily observed in older adults without dementia..

## Discussion

### Overview

This study explored the use of engagement-related behaviors demonstrated by older adults while playing serious computer games to provide valuable information about the presence of dementia. Our results support hypothesis 1, as 64% of engagement-related behaviors were significantly different between older adults with and without dementia. Using ML, key features distinguishing between older adults with and without dementia during serious gameplay include behaviors in categories of torso, voice, facial expression, concentration, and age, supporting hypothesis 2.

### Serious Gaming, Engagement-Related Patterns, and Dementia Differentiation

In our study, we observed engagement-related behaviors among older adults in behaviors such as directing attention toward the game, head scanning behavior or scanning the screen. These findings suggest that certain elements of engagement in gameplay are universal, regardless of an individual’s cognitive status. Similarly, Perugia et al [16] identified head movements as the most prominent engagement-related behavior in older adults, followed by torso movement, arm or hand movement, and gaze directed toward the activity when older adults with dementia interacted with a robot and played a board game.

The behaviors that were significantly different in frequency between the 2 groups, were also found to be important features to distinguish older adults with dementia from those without dementia. These behaviors are leaning forward torso behavior, upright torso behavior, voice utterances, lip behavior, not being distracted by external stimuli, and playing while doing something else. The present study observed that older adults with dementia demonstrated a higher percentage of upright torso behavior compared to older adults without dementia. Conversely, older adults without dementia exhibited a higher percentage of leaning forward torso behavior compared to those with dementia. Perugia et al [16] found that leaning forward was a common behavior in older adults with dementia. This discrepancy between Perugia et al’s [16] results and ours could be attributed to the use of different tasks. While Perugia et al [16] used board games that typically necessitate leaning forward, our study employed tablet-based games, allowing for a range of postures facilitated by tablet stands and varied grasps and angles of holding a tablet. It is known that the environment (eg, use of tablet stands) can influence engagement-related behaviors in older adults with dementia [16,26]. In our study, 80% of our participants in each group (ie, 4 out of 5 older adults living with dementia and 8 out of 10 older adults without dementia) decided to use tablet stands to angle the tablet screen to improve the view of the game. Therefore, in the present study, the environmental condition seems not to have impacted the torso behaviors of older adults, as they equally preferred to use the tablet stands to angle the tablet in the 2 groups. Another explanation could be that people living with dementia may experience increase anxiety when leaving home [27]. This could explain that our participants living with dementia felt over-attentive or less comfortable with the environment than older adults without dementia, resulting in sitting upright as a predominant behavior in participants with dementia.

Approximately one-third of the time intervals involved older adults with dementia exhibiting voiced utterances, whereas this finding was observed in less than 1% of the time intervals for older adults without dementia. Similarly, the results showed that older adults living with dementia had nearly twice the frequencies in lip behavior than older adults without dementia, suggesting that lip behaviors might be more common in older adults with dementia. These utterances, such as “oh,” “oops,” “um,” etc, and lip behavior (eg, mouthing words), may stem from changes in speech-language processes associated with dementia, impacting verbal communication [28,29].

The study revealed that engaging in concurrent activities while playing was more prevalent in older adults living with dementia compared to those without dementia. This finding suggests that individuals with dementia may be prone to distraction during gameplay, which can be attributed to cognitive decline, reduced ability to sustain focus, and impaired capacity to disregard distractors [26,30]. These findings emphasize the significance of considering the impact of external stimuli on engagement-related behaviors in individuals with dementia and underscore the necessity for tailored interventions that address attentional challenges.

The study revealed the absence of certain engagement-related behaviors in older adults with dementia compared to cognitively intact older adults, such as saccadic eye movements, play arm position adjustment, and keeping up with the game. Saccadic eye movements, rapid changes in gaze directions, appear to decline and become slower with cognitive impairment and dementia [31,32]. The lack of arm adjustment may reflect both the cognitive and perceptual-motor changes commonly associated with dementia, which can affect visuospatial skills and the ability to adjust body movements [33,34]. The inability to keep up with the game might signal a reduction in processing speed, a common cognitive deficit in dementia [35] in line with results from a separate serious game intervention for older adults with dementia by Tziraki et al [36] , as well as changes in vigilance, sustained attention, and fatigue [37].

### Classification Model of Dementia Status Using Serious-Gaming Engagement in Older Adults

This research further examined the potential of using ML to differentiate older adults with and without dementia based on their engagement-related behaviors while playing serious computer games. Model 4 emerged as the most effective to distinguish the presence of dementia based on engagement-related behaviors alongside personal, technical, and environmental factors. The identified engagement-related behaviors may aid in recognizing early signs of dementia, complementing standardized cognitive screening tools and information provided by older adults and their families.

Our study achieved similar accuracies to other studies that used ML to distinguish older adults with and without dementia [5,6]. The comparable accuracies achieved in existing ML studies suggest that playing mobile games can serve as a safe and cost-effective means of identifying older adults with and without dementia based on their engagement-related behaviors. This approach is less complex, risky, and costly compared to other approaches, such as using driving-related assessments, making it more accessible for older adults and their caregivers, particularly in rural or low-income settings.

### Limitations

This proof-of-concept study has several limitations that warrant consideration and guide future research. A primary limitation is the small sample size, which affects the accuracy and generalizability of the ML model’s predictions for older adults living with dementia. ML models typically require large and diverse datasets to achieve robust performance [38]. Future studies should aim to recruit larger and more diverse participant samples to enhance generalizability. Additionally, the potential for sample bias cannot be overlooked. Engagement behaviors are highly context-dependent and influenced by environmental factors, yet this study was conducted in only 2 settings. To address this, a multicenter approach, involving participants from various demographics, cultural backgrounds, and care environments, would provide a more comprehensive understanding of engagement-related behaviors and improve the applicability of the findings.

Despite the rigorous development of the coding system used to generate the dataset of engagement-related behaviors, some limitations persist. Strategies to minimize bias included a systematic 3-stage approach, rater training in engagement theory, and the use of independent raters. However, biases inherent to human judgment and the specific contexts of this study may still affect the coding system’s reliability and generalizability. To strengthen its validity, future research should test the coding system with different populations, raters, environments, and games. External validation across multiple research teams would further enhance its reliability and reproducibility.

Furthermore, the study highlights the need to explore engagement behaviors in diverse settings, such as home environments, community centers, and clinical care facilities. Investigating how context influences engagement will help refine ML models to account for environmental variability, making them more robust and broadly applicable.

Finally, while this study supports the potential of digital assessments, further work is needed to validate and standardize these tools. Ensuring their reliability, validity, and clinical utility for detecting cognitive decline in older adults remains a crucial step for their adoption in practice [3,39].

### Conclusions

Key features distinguishing between older adults with and without dementia during serious gameplay included torso, voice, facial, and concentration behaviors, as well as age. The best-performing ML model identified included features of engagement-related behaviors, environmental disturbances, technical challenges, and personal attributes. These findings have important implications for dementia research, suggesting opportunities for the ongoing development of engagement-targeted assessments and therapeutic interventions through the integration of serious gaming and ML.

## Supplementary material

10.2196/54797 Multimedia Appendix 1 Supplementary Table 1: *χ*² and Fisher exact tests.

## Abbreviations:

AD: Alzheimer disease
AUC: area under the receiver operating curve
ML: machine learning
RF: random forest
