# Supplementary material for: Machine Learning Analysis of Engagement Behaviors in Older Adults With Dementia Playing Mobile Games: Exploratory Study
Source: JMIR Serious Games. 2025 Mar 3;13:e54797. doi: 10.2196/54797 (PMC11892541; doi:10.2196/54797)
Supplement: Multimedia Appendix 1 [file games-v13-e54797-s001.docx]

**Supplementary Table 1: Chi-squared and Fisher’s exact tests**

| Category | Behaviour | Observation | Frequency of behaviours | | Total frequencies | Chi-square^a^ or Fisher’s exact test^b^ | |
| --- | --- | --- | --- | --- | --- | --- | --- |
|  |  |  | Dementia  (n = 591) | Without dementia  (n =1183) |  | Statistic | *P*-value |
| Gaze | Gaze towards game | Yes | 585 (99%) | 1,182 (99.9%) | 1,767 (99.6%) | 8.685 | .006^b^ |
|  |  | No | 6 (1%) | 1 (0.1%) | 7 (0.4%) |  |  |
|  |  | **Total** | 591 (100%) | 1,183 (100%) | 1,774 (100%) |  |  |
|  | Gaze away from the game | Yes | 42 (7.1%) | 68 (5.7%) | 110 (6.2%) | 1.250 | .263^a^ |
|  |  | No | 549 (92.9%) | 1,115 (94.3%) | 1,664 (93.8%) |  |  |
|  |  | **Total** | 591 (100%) | 1,183 (100%) | 1,774 (100%) |  |  |
| Eyes | Saccadic eye movements | Yes | 0 (0%) | 119 (10%) | 119 (6.7%) | 63.724 | < .001^b^ |
|  |  | No | 591 (100%) | 1,064 (90%) | 1,655 (93.3%) |  |  |
|  |  | **Total** | 591 (100%) | 1,183 (100%) | 1,774 (100%) |  |  |
|  | Eyes Scanning behaviours | Yes | 587 (99.3%) | 1,173 (99.1%) | 1,760 (99.2%) | 0.142 | 1.000^b^ |
|  |  | No | 4 (0.7%) | 10 (0.9%) | 14 (0.8%) |  |  |
|  |  | **Total** | 591 (100%) | 1,183 (100%) | 1,774 (100%) |  |  |
|  | Squinting at screen | Yes | 0 (0%) | 2 (0.2%) | 2 (0.1%) | 1.000 | .317^b^ |
|  |  | No | 591 (100%) | 1,181 (99.8%) | 1,772 (99.9%) |  |  |
|  |  | **Total** | 591 (100%) | 1,183 (100%) | 1,774 (100%) |  |  |
|  | Closed eyes | Yes | 1 (0.2%) | 2 (0.2%) | 3 (0.2%) | 4.775 | 1.000^b^ |
|  |  | No | 590 (99.8%) | 1,181 (99.8%) | 1,771 (99.8%) |  |  |
|  |  | **Total** | 591 (100%) | 1,183 (100%) | 1,774 (100%) |  |  |
|  | Eyes fixed on point | Yes | 8 (1.4%) | 0 (0%) | 8 (0.5%) | 16.086 | < .001^b^ |
|  |  | No | 583 (98.64%) | 1,183 (100%) | 1,766 (99.5%) |  |  |
|  |  | **Total** | 591 (100%) | 1,183 (100%) | 1,774 (100%) |  |  |
| Head | Head Scanning Behaviour | Yes | 587 (99.32%) | 1,178 (99.6%) | 1,765 (99.5%) | 0.504 | .491 |
|  |  | No | 4 (0.68%) | 5 (0.4%) | 9 (0.5%) |  |  |
|  |  | **Total** | 591 (100%) | 1,183 (100%) | 1,774 (100%) |  |  |
|  | Head learning toward game | Yes | 4 (0.6%) | 154 (13%) | 158 (8.9%) | 73.981 | < .001^b^ |
|  |  | No | 587 (99.4%) | 1,029 (87%) | 1,616 (91.1%) |  |  |
|  |  | **Total** | 591 (100%) | 1,183 (100%) | 1,774 (100%) |  |  |
|  | Head oriented away from the display | Yes | 25 (4.2%) | 34 (2.9%) | 59 (3.3%) | 2.254 | .159^a^ |
|  |  | No | 566 (95.8%) | 1,149 (97.1%) | 1,715 (96.7%) |  |  |
|  |  | **Total** | 591 (100%) | 1,183 (100%) | 1,774 (100%) |  |  |
| Torso | Leaning forward | Yes | 9 (1.5%) | 551 (46.6%) | 560 (31.6%) | 370.321 | < .001^a^ |
|  |  | No | 582 (98.5%) | 632 (53.4%) | 1,214 (68.4%) |  |  |
| Torso (continued) |  | **Total** | 591 (100%) | 1,183 (100%) | 1,774 (100%) |  |  |
|  | Upright posture | Yes | 589 (99.7%) | 636 (53.8%) | 1,225 (69%) | 388.545 | < .001^b^ |
|  |  | No | 2 (0.3%) | 547 (46.2%) | 549 (31%) |  |  |
|  |  | **Total** | 591 (100%) | 1,183 (100%) | 1,774(100%) |  |  |
|  | Slouched posture | Yes | 0 (0%) | 0 (0%) | 0 (0%) | - | - |
|  |  | No | 591 (100%) | 1,183 (100%) | 1,774 (100%) |  |  |
|  |  | **Total** | 591 (100%) | 1,183 (100%) | 1,774(100%) |  |  |
|  | Turned away from the game | Yes | 2 (0.3%) | 14 (1.2%) | 16 (1%) | 3.148 | .107^b^ |
|  |  | No | 589 (99.7%) | 1,169 (98.8%) | 1,758 (99%) |  |  |
|  |  | **Total** | 591 (100%) | 1,183 (100%) | 1,774 (100%) |  |  |
| Limbs | Limbs Scanning behaviour | Yes | 410 (69.4%) | 738 (62.4%) | 1,148 (64.7%) | 8.433 | .003^a^ |
|  |  | No | 181 (30.6%) | 445 (37.6%) | 626 (35.3%) |  |  |
|  |  | **Total** | 591 (100%) | 1,183 (100%) | 1,774 (100%) |  |  |
|  | Adjust the position of the tablet | Yes | 16 (2.7%) | 8 (0.7%) | 24 (1.3%) | 12.181 | < .001^a^ |
|  |  | No | 575 (97.3%) | 1,175 (99.3%) | 1,750 (98.7%) |  |  |
| Limbs (continued) |  | **Total** | 591 (100%) | 1,183 (100%) | 1,774 (100%) |  |  |
|  | Play hand ready | Yes | 462 (78.2%) | 1,049 (88.7%) | 1,511 (85.2%) | 34.411 | < .001^a^ |
|  |  | No | 129 (21.8%) | 134 (11.3%) | 263 (14.8%) |  |  |
|  |  | **Total** | 591 (100%) | 1,183 (100%) | 1,774 (100%) |  |  |
|  | Play arm position adjustment | Yes | 0 (0%) | 3 (0.3%) | 3 (0.2%) | 1.501 | .555^b^ |
|  |  | No | 591 (100%) | 1,180 (99.7%) | 1,771 (99.8%) |  |  |
|  |  | **Total** | 591 (100%) | 1,183 (100%) | 1,774 (100%) |  |  |
|  | Non-play hand at mouth | Yes | 5 (0.9%) | 136 (11.6%) | 141 (8%) | 61.098 | < .001^a^ |
|  |  | No | 586 (99.1%) | 1,047 (88.4%) | 1,633 (92%) |  |  |
|  |  | **Total** | 591 (100%) | 1,183 (100%) | 1,774 (100%) |  |  |
|  | Play hand away from device | Yes | 26 (4.4%) | 23 (2%) | 49 (2.8%) | 8.844 | .002^a^ |
|  |  | No | 565 (95.6%) | 1,160 (98%) | 1,725 (97.2%) |  |  |
|  |  | **Total** | 591 (100%) | 1,183 (100%) | 1,774 (100%) |  |  |
|  | Play hand movements unrelated to the game | Yes | 6 (1%) | 1 (0.1%) | 7 (0.4%) | 8.685 | .006^b^ |
|  |  | No | 585 (99%) | 1,182 (99.9%) | 1,767 (99.6%) |  |  |
|  |  | **Total** | 591 (100%) | 1,183 (100%) | 1,774 (100%) |  |  |
| Face | Smacking face | Yes | 0 (0%) | 2 (0.2%) | 2 (0.1%) | 1.000 | .317^a^ |
|  |  | No | 591 (100%) | 1,181 (99.8%) | 1,772 (99.9%) |  |  |
|  |  | **Total** | 591 (100%) | 1,183 (100%) | 1,774 (100%) |  |  |
|  | Neutral expression | Yes | 491 (83%) | 1,112 (94%) | 1,603 (90.4%) | 53.944 | < .001^a^ |
|  |  | No | 100 (17%) | 71 (6%) | 171 (9.6%) |  |  |
|  |  | **Total** | 591 (100%) | 1,183 (100%) | 1,774 (100%) |  |  |
|  | Lip behaviour | Yes | 347 (58.7%) | 373 (31.5%) | 720 (40.6%) | 120.775 | < .001^a^ |
|  |  | No | 244 (41.3%) | 810 (68.5%) | 1,054 (59.4%) |  |  |
|  |  | **Total** | 591 (100%) | 1,183 (100%) | 1,774 (100%) |  |  |
|  | Eyebrow movement | Yes | 24 (4%) | 310 (26.2%) | 334 (18.8%) | 126.448 | < .001^a^ |
|  |  | No | 567 (96%) | 873 (73.8%) | 1,440 (81.2%) |  |  |
|  |  | **Total** | 591 (100%) | 1,183 (100%) | 1,774 (100%) |  |  |
|  | Playful grimace | Yes | 6 (1%) | 7 (0.6%) | 13 (0.7%) | 0.971 | .324^a^ |
|  |  | No | 585 (99%) | 1,176 (99.4%) | 1,761 (99.3%) |  |  |
|  |  | **Total** | 591 (100%) | 1,183 (100%) | 1,774 (100%) |  |  |
|  | Open mouth | Yes | 8 (1.4%) | 72 (6%) | 80 (4.5%) | 20.498 | < .001^a^ |
| Face (continued) |  | No | 583 (98.6%) | 1,111 (94%) | 1,694 (95.5%) |  |  |
|  |  | **Total** | 591 (100%) | 1,183 (100%) | 1,774 (100%) |  |  |
|  | Surprised expression | Yes | 24 (4%) | 4 (0.3%) | 28 (1.6%) | 35.160 | < .001^b^ |
|  |  | No | 567 (96%) | 1,179 (99.7%) | 1,746 (98.4%) |  |  |
|  |  | **Total** | 591 (100%) | 1,183 (100%) | 1,774 (100%) |  |  |
|  | Smiling | Yes | 128 (21.7%) | 78 (6.6%) | 206 (11.6%) | 87.144 | < .001^a^ |
|  |  | No | 463 (78.3%) | 1,105 (93.4%) | 1,568 (88.4%) |  |  |
|  |  | **Total** | 591 (100%) | 1,183 (100%) | 1,774 (100%) |  |  |
|  | Tongue behaviour | Yes | 12 (2%) | 23 (2%) | 35 (2%) | 0.015 | .902^a^ |
|  |  | No | 579 (98%) | 1,160 (98%) | 1,739 (98%) |  |  |
|  |  | **Total** | 591 (100%) | 1,183 (100%) | 1,774 (100%) |  |  |
|  | Pained expression | Yes | 2 (0.3%) | 2 (0.2%) | 4 (0.3%) | 0.502 | .604^b^ |
|  |  | No | 589 (99.7%) | 1,181 (99.8%) | 1,770 (99.7%) |  |  |
|  |  | **Total** | 591 (100%) | 1,183 (100%) | 1,774 (100%) |  |  |
|  | Angry expression (e.g., baring teeth) | Yes | 0 (0%) | 25 (2.1%) | 25 (1.4%) | 12.667 | < .001^b^ |
| Face (continued) |  | No | 591 (100%) | 1,158 (97.9%) | 1,749 (98.6%) |  |  |
|  |  | **Total** | 591 (100%) | 1,183 (100%) | 1,774 (100%) |  |  |
|  | Frowning | Yes | 2 (0.3%) | 56 (4.7%) | 58 (3.3%) | 24.074 | < .001^b^ |
|  |  | No | 589 (99.7%) | 1,127 (95.4%) | 1,716 (96.7%) |  |  |
|  |  | **Total** | 591 (100%) | 1,183 (100%) | 1,774 (100%) |  |  |
|  | Yawning | Yes | 0 (0%) | 6 (0.5%) | 6 (0.4%) | 3.007 | .187^b^ |
|  |  | No | 591 (100%) | 1,177 (99.5%) | 1,768 (99.6%) |  |  |
|  |  | **Total** | 591 (100%) | 1,183 (100%) | 1,774 (100%) |  |  |
| Voice | Voiced utterance | Yes | 164 (27.7%) | 8 (0.7%) | 172 (9.7%) | 329.927 | < .001^a^ |
|  |  | No | 427 (72.3%) | 1,175 (99.3%) | 1,602 (90.3%) |  |  |
|  |  | **Total** | 591 (100%) | 1,183 (100%) | 1,774 (100%) |  |  |
|  | Voiceless utterance | Yes | 105 (17.8%) | 29 (2.5%) | 134 (7.5%) | 132.378 | < .001^a^ |
|  |  | No | 486 (82.2%) | 1,154 (97.5%) | 1,640 (92.5%) |  |  |
|  |  | **Total** | 591 (100%) | 1,183 (100%) | 1,774 (100%) |  |  |
| Voice (continued) | Laughing | Yes | 21 (3.5%) | 11 (1%) | 32 (1.9%) | 15.313 | < .001^a^ |
|  |  | No | 570 (96.5%) | 1,172 (99%) | 1,742 (98.1%) |  |  |
|  |  | **Total** | 591 (100%) | 1,183 (100%) | 1,774 (100%) |  |  |
|  | Frustrated exclamation | Yes | 3 (0.5%) | 22 (1.9%) | 25 (1.4%) | 5.185 | .030^b^ |
|  |  | No | 588 (99.5%) | 1,161 (98.1%) | 1,749 (98.6%) |  |  |
|  |  | **Total** | 591 (100%) | 1,183 (100%) | 1,774 (100%) |  |  |
|  | Refusal | Yes | 0 (0%) | 1 (0%) | 1 (0%) | 0.499 | 1.000^b^ |
|  |  | No | 591 (100%) | 1,182 (100%) | 1,773 100%) |  |  |
|  |  | **Total** | 591 (100%) | 1,183 (100%) | 1,774 100%) |  |  |
| Gameplay | Physical interaction with the screen | Yes | 574 (97.1%) | 1,181 (99.8%) | 1,755 (99%) | 27.265 | < .001^b^ |
|  |  | No | 17 (2.9%) | 2 (0.2%) | 19 (1%) |  |  |
|  |  | **Total** | 591 (100%) | 1,183 (100%) | 1,774 (100%) |  |  |
|  | Keeping up with the game | Yes | 0 (0%) | 86 (7.3%) | 86 (4.8%) | 45.152 | < .001^b^ |
|  |  | No | 591 (100%) | 1,097 (92.7%) | 1,688 (95.2%) |  |  |
|  |  | **Total** | 591 (100%) | 1,183 (100%) | 1,774 (100%) |  |  |
|  | Not physically interacting with the screen | Yes | 24 (4%) | 28 (2.4%) | 52 (3%) | 3.975 | .046^a^ |
|  |  | No | 567 (96%) | 1,155 (97.6%) | 1,722 (97%) |  |  |
| Gameplay (continued) |  | **Total** | 591 (100%) | 1,183 (100%) | 1,774 (100%) |  |  |
|  | Not keeping up with the game | Yes | 2 (0.3%) | 2 (0.2%) | 4 (0.2%) | 0.502 | .604^b^ |
|  |  | No | 589 (99.7%) | 1,181 (99.8%) | 1,770 (99.8%) |  |  |
|  |  | **Total** | 591 (100%) | 1,183 (100%) | 1,774 (100%) |  |  |
| Concentration | Not distracted by external stimuli | Yes | 433 (73.3%) | 1,178 (99.6%) | 1,611 (90.8%) | 326.994 | < .001^a^ |
|  |  | No | 158 (26.7%) | 5 (0.4%) | 163 (9.2%) |  |  |
|  |  | **Total** | 591 (100%) | 1,183 (100%) | 1,774 (100%) |  |  |
|  | Playing while doing something else | Yes | 149 (25.2%) | 5 (0.4%) | 154 (8.7%) | 5.494 | < .001^a^ |
|  |  | No | 442 (74.8%) | 1,178 (99.6%) | 1,620 (91.3%) |  |  |
|  |  | **Total** | 591 (100%) | 1,183 (100%) | 1,774 (100%) |  |  |
|  | Stopping play to attend to another stimulus | Yes | 42 (7.1%) | 62 (5.2%) | 104 (5.9%) | 2.485 | .114^a^ |
|  |  | No | 549 (92.9%) | 1,121 (94.8%) | 1,670 (94.1%) |  |  |
|  |  | **Total** | 591 (100%) | 1,183 (100%) | 1,774 (100%) |  |  |
| Breath | Rhythmic breathing | Yes | 289 (48.9%) | 298 (25.2%) | 587 (33%) | 100.068 | < .001^a^ |
|  |  | No | 302 (51.1%) | 885 (74.8%) | 1,187 (67%) |  |  |
|  |  | **Total** | 591 (100%) | 1,183 (100%) | 1,774 (100%) |  |  |

*Notes: ^a^* Chi-squared test; ^b^ Fisher’s exact test.
